# Postoperative computed tomography imaging of pediatric patients with craniosynostosis: radiation dose and image quality comparison between multi-slice computed tomography and O-arm cone-beam computed tomography

**DOI:** 10.1007/s00247-023-05644-3

**Published:** 2023-03-27

**Authors:** Touko Kaasalainen, Ville Männistö, Teemu Mäkelä, Juho Suojanen, Antton Nuorala, Arja Heliövaara, Junnu Leikola

**Affiliations:** 1 HUS Diagnostic Center, Radiology, University of Helsinki and Helsinki University Hospital, P.O. Box 340, 00290 Helsinki, Finland; 2 Department of Oral and Maxillofacial Surgery, Lahti Central Hospital, Päijät-Häme Joint Authority for Health and Wellbeing, Lahti, Finland; 3 Department of Physics, University of Helsinki, Helsinki, Finland; 4 Cleft Palate and Craniofacial Center, Department of Plastic Surgery, Helsinki University Hospital and University of Helsinki, Helsinki, Finland; 5 Faculty of Medicine, University of Helsinki, Helsinki, Finland

**Keywords:** Computed tomography, Cone-beam computed tomography, Craniosynostosis, Imaging, O-arm, Pediatric, Skull

## Abstract

**Background:**

When postoperative multi-slice computed tomography (MSCT) imaging of patients with craniosynostosis is used, it is usually performed a few days after surgery in a radiology department. This requires additional anesthesia for the patient. Recently, intraoperative mobile cone-beam CT (CBCT) devices have gained popularity for orthopedic and neurosurgical procedures, which allows postoperative CT imaging in the operating room.

**Objective:**

This single-center retrospective study compared radiation dose and image quality of postoperative imaging performed using conventional MSCT scanners and O-arm CBCT.

**Materials and methods:**

A total of 104 pediatric syndromic and non-syndromic patients who were operated on because of single- or multiple-suture craniosynostosis were included in this study. The mean volumetric CT dose index (CTDI_vol_) and dose-length product (DLP) values of optimized craniosynostosis CT examinations (58 MSCT and 46 CBCT) were compared. Two surgeons evaluated the subjective image quality.

**Results:**

CBCT resulted in significantly lower CTDI_vol_ (up to 14%) and DLP (up to 33%) compared to MSCT. Multi-slice CT image quality was considered superior to CBCT scans. However, all scans were considered to be of sufficient quality for diagnosis.

**Conclusion:**

The O-arm device allowed for an immediate postoperative CBCT examination in the operating theater using the same anesthesia induction. Radiation exposure was lower in CBCT compared to MSCT scans, thus further encouraging the use of O-arms. Cone-beam CT imaging with an O-arm is a feasible method for postoperative craniosynostosis imaging, yielding less anesthesia to patients, lower health costs and the possibility to immediately evaluate results of the surgical operation.

## Introduction

Craniosynostosis is a disturbance in head and skull development in which premature fusion of one or more cranial sutures results in abnormal skull growth [1, 2]. The prevalence of craniosynostosis is 3.1 to 7.2 per 10,000 live births [3–5]. Early diagnosis and surgical management are essential for craniosynostosis patients to achieve acceptable clinical outcomes and to reduce the number of recurrent surgical interventions. Surgical procedures allow the brain to grow and expand normally and improve the esthetics and function of the craniofacial region [6]. Untreated craniosynostosis typically leads to ongoing deformity and sometimes an increase in intracranial pressure with possible neurological consequences [2, 7].

Currently, multi-slice computed tomography (MSCT) imaging is widely used together with cranial X-ray imaging, ultrasound and magnetic resonance imaging (MRI) for the diagnosis and follow-up of craniosynostosis patients. The sutures of the calvaria and the skull base are most accurately identified on axial and 3-dimensional (D) surface-rendered CT images, which is currently considered the gold standard in the radiological diagnosis of craniosynostosis [8–10]. Preoperative CT images are used to confirm clinical diagnosis, assess structural anomalies and neurovascular anatomy and facilitate accurate surgical planning [10, 11]. Postoperatively, CT images can be used to detect possible complications and evaluate the surgical outcome and adequacy of calvarial expansion [10, 11]. Additionally, postoperative evaluation is beneficial for the assessment of new surgical techniques and in medicolegal disputes. As the contrast of the skull compared to the cranial soft tissues is higher than the contrast between cranial soft tissues, CT examinations of the skull can be performed with a significantly lower radiation level than that of routine head CT scans. This is highly important as the entire diagnostic chain in craniosynostosis typically includes repeated CT examinations (pre- and postoperative) with associated exposure to ionizing radiation, which is harmful to health [12, 13]. In addition to longer life expectancy, children are also more radiosensitive than adults, which increases the requirement for dose optimization [14, 15]. Depending on the level of exposure, radiation from CT imaging increases brain cancer risk in children up to five fold [16]. When postoperative CT scans for craniosynostosis are used, these are typically performed a few days after surgery in the radiology department. This requires an additional anesthesia for the patient, which increases healthcare costs and risks. Recently, 3-D intraoperative cone-beam CT (CBCT) imaging performed either with a mobile C-arm or an O-arm has become increasingly common, especially for orthopedic and neurosurgical procedures. This provides possibilities not only for intraoperative but also for postoperative CT scans in the operating theater [17–23].

Replacing a conventional MSCT examination performed in the radiology department with a time- and cost-effective CBCT examination performed with a mobile intraoperative O-arm system in the surgical department offers multiple benefits. This study aimed to assess the feasibility of postoperative CBCT by evaluating the differences in image quality and radiation dose between the two CT techniques.

## Materials and methods

This single-center retrospective study included 104 pediatric syndromic and non-syndromic patients who were operated on because of single- or multiple-suture craniosynostosis at Helsinki University Hospital between January 2014 and July 2020. All patients were examined with preoperative head CT, MRI or both before the procedure. The postoperative CT scans were performed either with a conventional MSCT scanner (see next section) in a radiology department (*n*=58) or with a mobile intraoperative O-arm CBCT system (Medtronic, Minneapolis, MN) in a surgical department (*n*=46). The study was approved by the Ethics Committee of the Hospital District of Helsinki and Uusimaa (HUS/221/2017 §47) and was performed according to the principles outlined in the Declaration of Helsinki. Because of the retrospective nature of the study, no informed patient consent was required. Individual-level data cannot be shared openly due to restrictions imposed by the research permit.

### Postoperative computed tomography scans performed with multi-slice computed tomograhy machines

The postoperative MSCT head examinations were performed in a head-first supine position using the following four CT systems: two 64-slice GE LightSpeed VCT (GE Healthcare, Milwaukee, WI), a 64-slice GE Discovery CT750 HD (GE Healthcare), and a 256-slice GE Revolution CT (GE Healthcare). The scan ranges of the postoperative CT examinations were typically adjusted to cover the patient anatomy from the bottom of the chin to the top of the head. Low-dose helical scan protocols developed and optimized for craniosynostosis imaging were used for all MSCT systems. All the scanners utilized a dynamic collimation mechanism for helical CT scans to prevent overranging and increased dose. The device-specific scanning parameters are presented in Table 1. For one postoperative CT examination with a GE LightSpeed VCT system, a routine axial head CT scan protocol for children was unintentionally used. This CT examination, with a mean volumetric CT dose index (CTDI_vol_) value of 28.67 mGy and dose-length product (DLP) of 466.6 mGy·cm, was excluded from further dose evaluations. For the GE Revolution CT, an organ-dose modulation technique (ODM, GE Healthcare, Milwaukee, WI) was used to reduce radiation exposure to the patient’s eye lens. Axial images were reconstructed using either adaptive statistical iterative reconstruction (ASiR) or ASiR-V with 20% to 40% ASiR-FBP (filtered back projection) blending. The following two sets of axial images were reconstructed: 0.625mm and 1.25mm thicknesses with soft and bone detail reconstruction filters, respectively. The image matrix was 512 × 512 and the display field of view (FOV) in the image plane was 20 × 20 cm^2^ or 22 × 22 cm^2^, resulting in voxel sizes of 0.39 × 0.39 × 0.63 mm^3^ or 0.43 × 0.43 × 0.63 mm^3^ for patients ≤18 months old and >18 months old, respectively. In addition to the axial images, 3-D surface-rendered images were created from the 0.625 mm soft reconstruction kernel images on a multi-modality image processing workstation (GE Advanced Workstation, version 4.4, Milwaukee, WI).

**Table 1:** Device-specific scanning parameters used for helical head computed tomography examinations of craniosynostosis patients

CT device	Detector configuration	Rotation time (s)	Pitch	Tube voltage (kVp)	NI (min–max mA)
GE LightSpeed VCT #1	64 × 0.625 mm	0.5	0.984	100 (≤ 18 months) and 120 (> 18 months)	33 (10–80 mA for children ≤ 18 months and 25–120 mA for children > 18 months)
GE LightSpeed VCT #2	32 × 0.625 mm	0.5	0.969	100 (≤ 18 months) and 120 (> 18 months)	33 (10–80 mA for children ≤ 18 months and 25–120 mA for children > 18 months)
GE Discovery CT750 HD	64 × 0.625 mm	0.5	0.984	100 (≤ 18 months) and 120 (> 18 months)	35 (10–80 mA for children ≤ 18 months and 25–120 mA for children > 18 months)
GE Revolution CT	64 × 0.625 mm	0.28	0.984	100	24 (10–250 mA)

*CT*, computed tomography; *NI*, noise index (an operator-selectable parameter to determine the desired noise level in GE CT systems (NI for GE Revolution CT is not comparable to the other GE systems used in this study])

### Postoperative cone-beam computed tomography scans performed with an O-arm device

The O-arm is a mobile intraoperative CBCT device designed for both 2-D and 3-D imaging. The equipment is specifically optimized for imaging of bony structures in spinal and orthopedic surgery, but has also been widely used for neurosurgical procedures of the brain [17, 19–23]. The CBCT system used in this study was a second-generation O-arm with a 40 cm × 30 cm amorphous silicon flat-panel detector and a conventional X-ray tube. For 3-D scanning of the head with a standard operating mode, a total of 196 2-D projection images were acquired during a 360° rotation with a rotation time of 13 s. The exposure time per rotation was 3.9 s due to pulsed irradiation. The 3-D scans were performed in a head-first supine position as a final step of cranial surgery. A Magnus operating table system (Maquet, Rastatt, Germany) with carbon-fiber tabletops was used for the procedures. The scan range was set using lateral and posterior-to-anterior fluoroscopic images to cover the entire skull. The FOV edge was aligned to cover the top of the head. The tube current (10 mA in all scans) and tube voltage were set manually instead of using predefined 3-D scan protocols from the vendor. The tube voltage was set to 70 kVp for patients <2-years-old, 80 kVp for patients from 2–10-years-old and 90 kVp for patients >10-years-old. The CBCT voxel size provided by the equipment was 0.42 × 0.42 × 0.83 mm^3^. In addition to the axial images, 3-D volume-rendered images were reconstructed either on IMPAX Volume Viewing 3-D clinical application tool (Agfa HealthCare, Mortsel, Belgium, version 6.6) or on the GE Advanced Workstation.

### Dose comparisons

The volumetric CT dose index (CTDI_vol_) and dose-length product  (DLP) values for each examination were retrospectively retrieved from the dose reports. Both the O-arm and the MSCT devices used a 16-cm reference head phantom for calculating and expressing the dose indices. The dose display accuracies of the systems were confirmed from the maintenance reports. The CTDI_vol_, DLP and tube voltages used in the postoperative CT scans were compared between the MSCT devices and between techniques (i.e, MSCT vs. CBCT). The radiation doses in terms of kerma-area product (KAP) of the O-arm 2-D lateral and posterior-to-anterior (fluoroscopic) planning images were also obtained from the dose reports.

### Subjective image quality

Two board-certified surgeons, a plastic surgeon (surgeon 1 [J.L.] with 18 years of experience) and an oral and maxillofacial surgeon (surgeon 2 [J.S.] with 8 years of experience), examined the reconstructed images independently in a blinded manner using a pair of digital imaging and communications in medicine (DICOM)-calibrated diagnostic monitors. The combination of general image quality appearance (noise, contrast and sharpness) and appropriateness of the images for confident diagnosis of each patient examination were evaluated using a 5-point Likert scale (1, extremely poor and clinically useless image quality, defined as excessive image noise, poor contrast and image sharpness preventing delineation of critical anatomical structures; 2, poor to suboptimal but still diagnostic image quality, defined as somewhat poor contrast and sharpness and extensive but still tolerable image noise for the indication; 3, suboptimal but diagnostic image quality, defined as tolerable contrast, sharpness and noise; 4, good image quality, defined as good contrast and sharpness and very little noise for the indication; 5, excellent image quality, defined as good contrast, excellent sharpness and little to no image noise). Moreover, from the whole 3-D image stack, the presence of image artefacts, 3-D image quality and interpretation of both orbital and skull base structures were each separately evaluated using a 3-point Likert scale (1, insufficient image quality, images do not allow diagnostic interpretation; 2, limited but still tolerable image quality, moderate image artefacts; 3, good image quality, no artefacts).

### Statistical analysis

Numeric results are presented as mean ± standard deviation where appropriate. Variable comparisons between the systems were performed using an independent sample Kruskal–Wallis test with a post hoc Bonferroni correction. Mann–Whitney *U* test was used in pairwise comparisons of MSCT (all MSCT systems combined) and CBCT (O-arm). Inter-rater reliability was evaluated using a linearly weighted Cohen’s *κ* correlation between the two readers. All statistical tests were two-sided, and a *P*-value < 0.05 was considered statistically significant. Statistical analyses were performed using SPSS statistical software (IBM, Armonk, NY, version 25.0).

## Results

### Baseline characteristics

A total of 104 pediatric syndromic and non-syndromic patients (mean age 1.8 ± 2.2 years, range 0.3–10.4 years) who were operated on because of single- or multiple-suture craniosynostosis were included in the study. No significant differences in patient age on day of surgery (1.6 ± 1.7 years and 2.0 ± 2.7 years; *P*=0.88) or sex (*n*=40 males (69%) and *n*=36 males (78%); *P*=0.29) were observed between the MSCT and CBCT groups.

### Dose comparisons

**Fig. 1:**
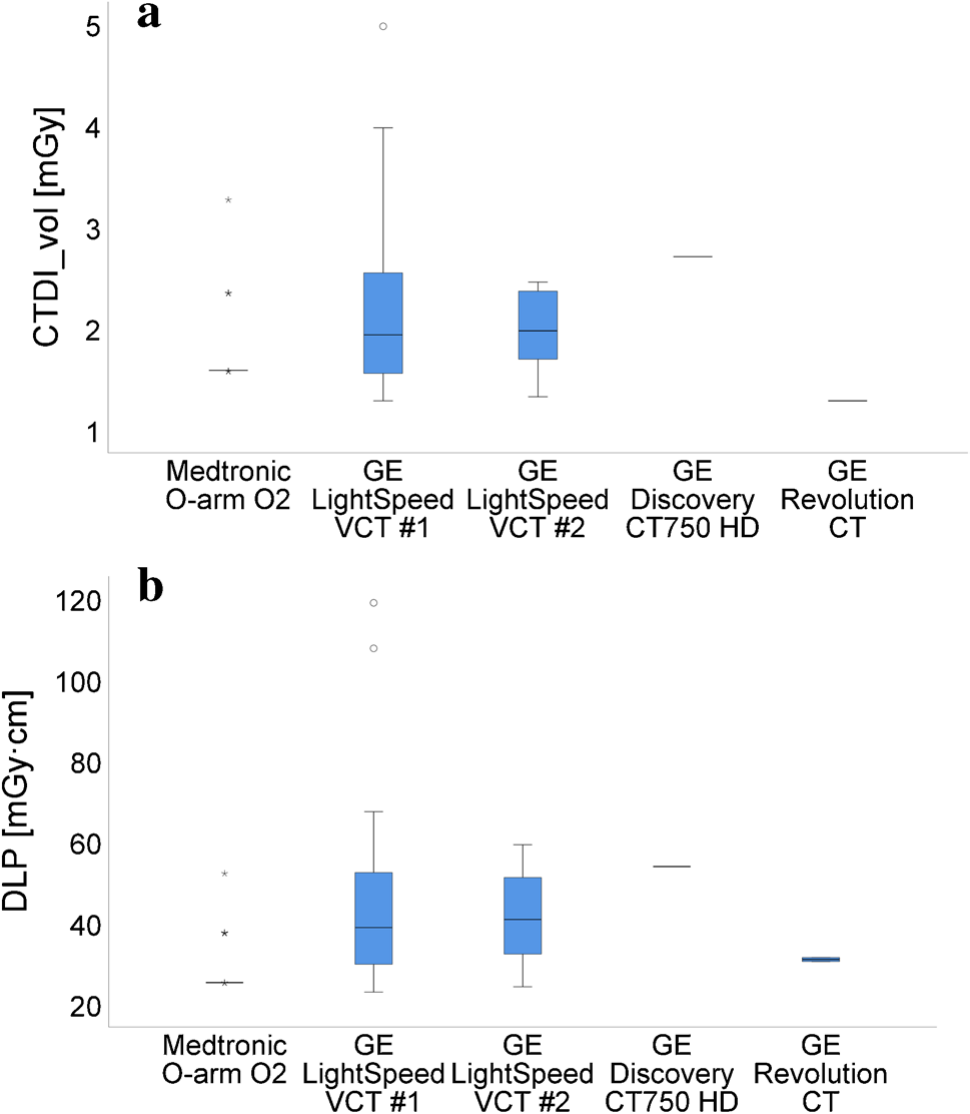
Boxplots showing the mean volumetric computed tomography dose index (CTDI_vol_) (**a**) and dose-length product (DLP) values (**b**) for the postoperative computed tomography (CT) scans of craniosynostosis patients with different 3-dimensional imaging systems

The dose display accuracies of all imaging systems were within ± 10% for the X-ray tube voltage ranges used in the patient examinations. Figure 1 and Table 2 summarize the CTDI_vol_ and DLP values of the postoperative CT scans. The low number of patients scanned with the GE Discovery CT750 HD (*n*=1) and GE Revolution CT (*n*=2) systems prevented a reliable statistical analysis of these devices. The mean CTDI_vol_ was up to 16% lower with the O-arm than with the GE LightSpeed VCT devices. Nevertheless, pairwise comparisons with Bonferroni correction did not reveal significant differences in the measured CTDI_vol_ values between the five systems. However, the O-arm device resulted in significantly lower DLP values (up to 34%) compared to GE Revolution LightSpeed VCT systems (*P* ≤ 0.002), reflecting a shorter craniocaudal scan range used with the O-arm system. The pairwise comparisons of the postoperative 3-D imaging techniques revealed significant differences in the mean CTDI_vol_ (1.79 ± 0.39 mGy vs. 2.09 ± 0.70 mGy; *P*=0.022) and DLP (28.62 ± 6.21 mGy·cm vs. 42.71 ± 18.23 mGy·cm; *P* < 0.001) values between the CBCT and MSCT techniques. The O-arm system used significantly lower tube voltages (70 kVp in 35/46 [76.1%] of the patients) for 3-D imaging than the MSCT systems (*P* < 0.001). Lower variation in dose indices were seen with the O-arm device than with the MSCT scanners utilizing tube current modulation. In O-arm 3-D scans, only the selected tube voltage affected patient exposure, as the tube current was manually set and was constant for all scans. In a single patient scanned with the O-arm, the CBCT scan with equal imaging parameters and radiation dose was performed twice due to improperly set FOV for the first scan (FOV did not reach the top of the skull). This doubled the exposure for this patient. The mean KAP from the fluoroscopy-guided FOV planning step prior to the O-arm CBCT scan was 400.5 ± 361.6 mGy·cm^2^.

**Table 2:** The mean volumetric computed tomography dose index (CTDI_vol_) and dose-length product (DLP) values of each imaging system for postoperative craniosynostosis 3-dimensional imaging

Dose index	Medtronic O-arm	GE LightSpeed VCT #1	GE LightSpeed VCT #2	GE Discovery CT750 HD	GE Revolution CT^a^	*P*-value	*P*-value < 0.05 between
*n* (%)	46 (44.2)	41 (39.4)	14 (13.5)	1 (1.0)	2 (1.9)		
CTDI_vol_ (mGy)	1.79 ± 0.39	2.13 ± 0.78	1.95 ± 0.38	2.71	1.29	0.040	^b^
DLP (mGy·cm)	28.62 ± 6.21	43.50 ± 20.24	41.14 ± 12.09	53.99	31.07 ± 0.68	< 0.001	O-arm vs. VCT #1 and #2

*CT, *computed tomography;* CTDI*_*vol*_, volumetric computed tomography dose index; *DLP*, dose-length product

^a^The dose report of a single examination was not sent to the Picture Archiving and Communication System from the scanner console and the CTDI_vol_ was therefore not available (the DLP for this scan was obtained from the patient’s electronic medical record)

^b^None of the pairwise comparisons remained significant after Bonferroni adjustment

The dose values are given as mean ± standard deviation

### Subjective image quality

**Fig. 2:**
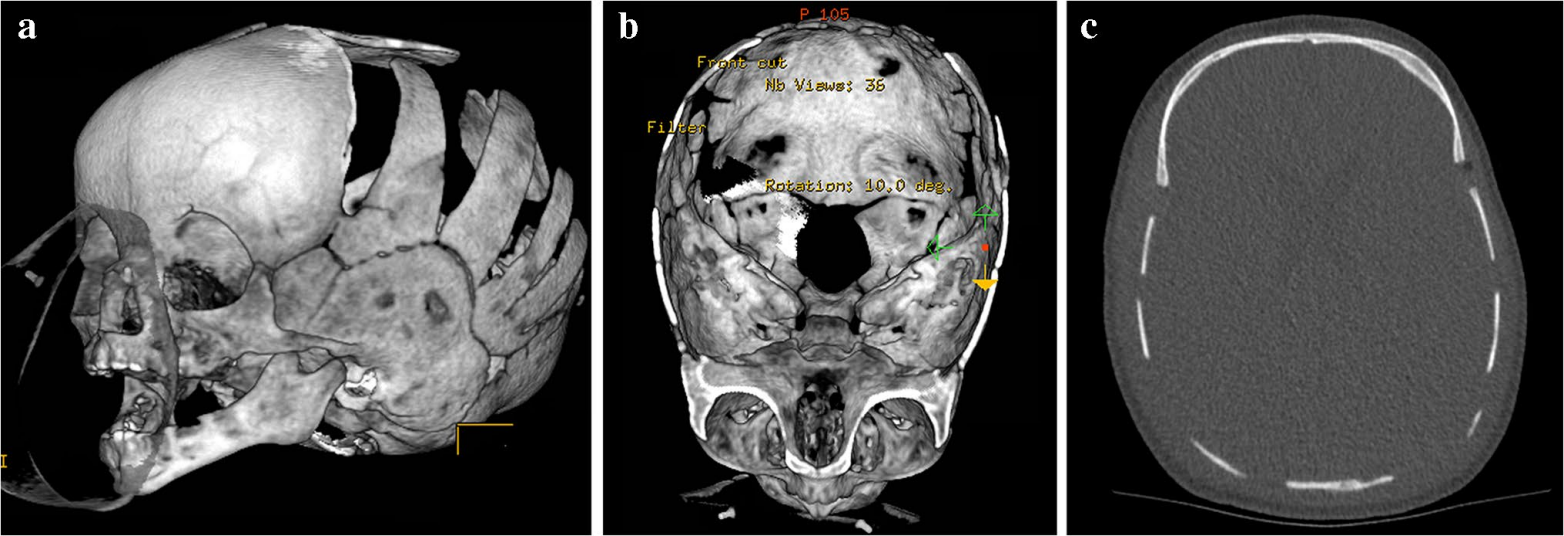
Postoperative non-contrast-enhanced computed tomography (CT) images of a 4-month-old boy with sagittal craniosynostosis.  Three-dimensional surface-rendered (**a**) intracranial surface-rendered (**b**) and axial (**c**) CT images clearly show the cranial sutures. Even though a low-dose CT protocol was used, the conventional multi-slice CT shows the ventricles due to sufficient soft tissue contrast of the imaging technique

**Fig. 3:**
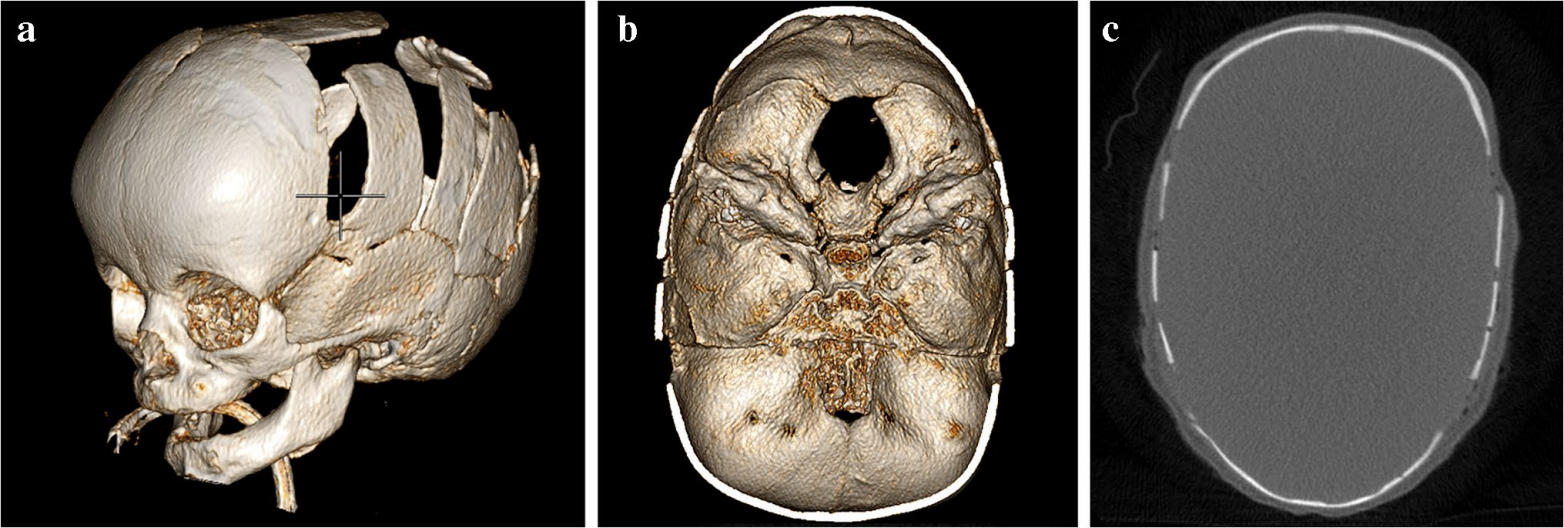
Postoperative non-contrast-enhanced cone-beam computed tomography (CBCT) images of a 5-month-old boy with sagittal craniosynostosis. Three-dimensional surface-rendered (**a**) intracranial surface-rendered (**b**) and axial (**c**) images clearly show the cranial sutures. However, the low-dose CBCT images did not have sufficient soft tissue contrast to show the ventricles

Examples of postoperative axial and surface-rendered 3-D images from a MSCT system and from the CBCT device are shown in Fig. 2 and Fig. 3, respectively. The conventional MSCT systems resulted in significantly higher subjective image quality ratings for all evaluated criteria compared with the CBCT. The ratings were 4.11 ± 0.65 and 4.91 ± 0.21 (*P* < 0.001) for general image quality (image noise, contrast and sharpness), 2.36 ± 0.69 and 2.72 ± 0.33 (*P* = 0.018) for image artefacts, 2.68 ± 0.43 and 2.95 ± 0.15 (*P* < 0.001) for 3-D image quality, 2.29 ± 0.75 and 2.98 ± 0.09 (*P* < 0.001) for the interpretation of orbital structure and 2.38 ± 0.63 and 2.96 ± 0.17 (*P* < 0.001) for the interpretation of skull base structures for the CBCT and MSCT, respectively. Neither surgeon rated general image quality insufficient for diagnoses for any of the scans. However, insufficient image quality was observed with the O-arm when evaluating the entire 3-D image stack for the presence of image artefacts (9/46 and 5/46 examinations [surgeon 1 and surgeon 2, respectively]), 3-D image quality (0/46 and 1/46 examinations), interpretation of orbital structures (7/46 and 11/46 examinations) and interpretation of skull base structures (4/46 and 4/46 examinations). The non-diagnostic areas in the images were not relevant for the required postoperative diagnoses.

The inter-rater reliability between the two surgeons varied from moderate to almost perfect depending on the evaluated criteria. The linearly weighted Cohen’s *κ* values were 0.53 (95% confidence interval [CI], 0.39 to 0.66; *P* < 0.001) for general image quality, 0.42 (95% CI, 0.26 to 0.58; *P* < 0.001) for image artefacts, 0.44 (95% CI, 0.39 to 0.66; *P* < 0.001) for 3-D image quality, 0.80 (95% CI, 0.68 to 0.91; *P* < 0.001) for the interpretation of orbital structures and 0.83 (95% CI, 0.72 to 0.95; *P* < 0.001) for the interpretation of skull base structures.

## Discussion

Postoperative CT imaging of patients with craniosynostosis is often performed a few days after surgery in the radiology department using MSCT. This usually requires additional anesthesia and thus increases both the risks to the patient and healthcare costs. From the perspectives of patient safety and economics, it would be beneficial to perform postoperative cranial CT examinations in the operating theater, although radiation dose and image quality may differ from that of MSCT examinations. This study aimed to verify a time- and cost-effective method of performing postoperative CBCT scans for craniosynostosis by replacing MSCT examinations with O-arm CBCT scans performed in the operating theater. The radiation dose and subjective image quality were compared between the methods.

Using an intraoperative O-arm CBCT imaging system for postoperative 3-D imaging can improve patient safety, as a second induction of anesthesia for the patient is not required. Moreover, the surgeon may reposition the skull structures immediately after the intraoperative 3-D imaging. In extreme cases of severe displacement of bony structures, an immediate correction can be performed avoiding, e.g., compression and subdural hemorrhage. The results of this retrospective study show that economic savings are also possible not only due to the reduced number of anesthesia inductions required but also because no manpower or equipment resources from radiology or other departments are required for the postoperative CT.

Patient radiation exposure from the CBCT scans was comparable to that of the MSCT scanners. Pairwise analysis revealed significantly lower mean CTDI_vol_ (1.79 ± 0.39 mGy vs. 2.09 ± 0.70 mGy; *P* = 0.022) and DLP (28.62 ± 6.21 mGy·cm vs. 42.71 ± 18.23 mGy·cm; *P* < 0.001) values for the CBCT compared to the MSCT protocol. The CBCT scan FOV was adjusted to cover the entire skull. For the MSCT scans performed in the radiology department, the scan range was usually adjusted to cover patient anatomy from the bottom of the chin to the top of the head (retrospectively it was found that there were two user instructions for postoperative MSCT scans, which were contradictory in terms of setting the scan range: one was correctly instructing the scan range to be from the upper jaw to the top of the head, while the other instructed the scan range to be from the bottom of the chin to the top of the head—the latter instruction was unfortunately followed more often). Thus, the scan range was typically longer for the MSCT examinations than for the CBCT scans, which increased the DLP. However, for purposes of postoperative imaging, covering the skull from the upper jaw to the top of the head typically provides sufficient information for the surgeon. Dose savings could therefore be achieved by limiting the MSCT scan length.

The subjective image quality of MSCT examinations was superior to that of CBCT scans. Generally, CBCT produces more image noise and lower soft tissue contrast than fan-beam CT technology used in MSCT systems [24–27]. In this study, the conventional MSCT technique especially improved the interpretation of orbital and skull base structures and reduced the number of image artefacts. Regardless, image quality was also considered sufficient for the craniosynostosis indication in all CBCT scans. Based on our image quality results, MSCT doses can be lowered even further to follow the ALARA principle (as low as reasonably achievable).

For craniosynostosis patients, a relatively high accumulated radiation dose for a child is common due to repeated CT examinations at the time of diagnosis and at various stages of surgical correction. The effective dose from a standard head CT varies from 0.5 mSv to 2 mSv. However, due to the inherent contrast of the skull, a CT examination when suspected craniosynostosis is the indication may be performed with a significantly lower exposure level. Previous studies have reported effective doses from craniosynostosis MSCT to vary widely from 0.02 mSv to 2.8 mSv [28–33]. By using the age-specific conversion factors given by Deak et al. (2010) [15] to determine effective doses from DLP values (mean *k* of 0.0087, 0.0054, 0.0035, and 0.0027 mSv/[mGy·cm] for newborn, 1-year-old, 5-year-old and 10-year-old children, respectively), the craniosynostosis patients in this retrospective study were exposed to effective doses ranging from 0.08 mSv to 0.27 mSv for the O-arm CBCT scans and from 0.12 mSv to 0.40 mSv for the MSCT scans. Notably, the KAP in the 2-D fluoroscopy mode to plan the CBCT scan FOV varied between patients (400.5 ± 361.6 mGy·cm^2^). By using conversion factors from KAP to effective dose (0.034 mSv/[Gy·cm^2^], 0.37 mSv/[Gy·cm^2^] and 0.058 mSv/[Gy·cm^2^] for posterior-to-anterior, lateral and anterior-to-posterior head radiographs, respectively) from Wall et al. (2011) [34], fluoroscopy resulted in an effective dose of 0.01 mSv to 0.04 mSv. Note that the conversion factors depend not only on patient age but also, for example, on the X-ray tube voltage and filtration. Thus, the effective doses reported above are only approximations and should be adopted with caution.

This study has some limitations. First, the total number of patients was small. Specifically, the small number of patients imaged with two of the four MSCT systems prevented device-specific analysis. However, the reported dose indices and scan parameters were comparable between the CT devices, and therefore, it is assumed that no remarkable differences occurred. Second, the study results are not based on a comparison of a postoperative MSCT and CBCT in the same patient. This would, however, not have been ethically justifiable. Third, the MSCT systems being studied were limited to only one manufacturer and a few models. As CT optimization methods and scan geometries differ between vendors (and between CT models from the same vendor), the conclusions from the scan protocols used here cannot be directly generalized to other MSCT systems. Moreover, older versions of GE CT scanners might also have influenced the MSCT doses negatively as detectors, iterative reconstructions etc. have improved and reduced doses. For example, deep-learning image reconstructions, such as GE’s TrueFidelity, may reduce patient doses even further, while maintaining sufficient image quality for diagnosis [35]. Fourth, only two surgeons evaluated the subjective image quality and radiologist raters were not used. This decision was made as, in our hospital, surgeons report the postoperative O-arm CBCT scans performed in our surgical unit. Pediatric radiologists are no longer involved in the immediate postoperative 3-D imaging of patients with craniosynostosis unless a CT scan needs to be performed in the radiology department due to O-arm equipment failure or lack of qualified O-arm users working on the shift in the surgery department (pediatric radiologists report the preoperative MSCT scans performed in the radiology department). Finally, image quality was only analyzed subjectively. Objective image quality analysis with, for example, more comprehensive image noise, contrast-to-noise, spatial resolution and noise power spectra analysis is likely to have revealed more differences between the methods. For example, it is commonly known that CBCT systems, which are designed specifically for imaging of bony structures, generate more image noise than MSCT systems. However, this study was mainly designed to determine the feasibility and clinical applications of the O-arm device for postoperative craniosynostosis CT imaging. In the future, phantom measurements with more comprehensive image quality analysis may be performed to further optimize the scan protocols.

## Conclusion

The patient radiation doses of the craniosynostosis imaging protocols studied were comparable between the CBCT and MSCT systems, with slightly lower doses reported for the former. Based on a subjective evaluation considering image noise, contrast, sharpness, presence of artefacts, 3-D image quality and interpretability of orbital and skull base structures, the CBCT technique showed a lower but diagnostic image quality compared to MSCT for postoperative CT imaging of patients with craniosynostosis. Thus, CBCT imaging using an O-arm device is a feasible option and may replace conventionally performed MSCT examinations for this indication. The most important benefits of intraoperative CBCT include less anesthesia, lower health costs and the possibility of evaluating the immediate postoperative results.

## Data Availability

Individual-level data cannot be shared openly due to restrictions imposed by the research permit.
